# Investigations on Transgenerational Epigenetic Response Down the Male Line in F2 Pigs

**DOI:** 10.1371/journal.pone.0030583

**Published:** 2012-02-16

**Authors:** Martin Braunschweig, Vidhya Jagannathan, Andreas Gutzwiller, Giuseppe Bee

**Affiliations:** 1 Institute of Genetics, Vetsuisse Faculty, University of Bern, Berne, Switzerland; 2 Federal Research Station for Animal Production, Agroscope Liebefeld-Posieux, Posieux, Switzerland; Massachusetts General Hospital, United States of America

## Abstract

We investigated the nutritional effects on carcass traits, gene expression and DNA methylation in a three generation Large White pig feeding experiment. A group of experimental (E) F0 boars were fed a standard diet supplemented with high amounts of methylating micronutrients whereas a control group (C) of F0 boars received a standard diet. These differentially fed F0 boars sired F1 boars which then sired 60 F2 pigs. Carcass traits were compared between 36 F2 descendants of E F0 boars and 24 F2 descendants of C F0 boars. The two F2 offspring groups differed with respect to backfat percentage (*P* = 0.03) and tended to differ with respect to adipose tissue (*P* = 0.09), fat thickness at the 10^th^ rib (*P* = 0.08) and at the croup (*P* = 0.09) as well as percentages of shoulder (*P* = 0.07). Offspring from the experimental F0 boars had a higher percentage of shoulder and were leaner compared to the control group. Gene expression profiles showed significant twofold differences in mRNA level between 8 C F2 offspring and 8 E F2 offspring for 79, 64 and 53 genes for muscle, liver and kidney RNA, respectively. We found that in liver and muscle respective pathways of lipid metabolism and metabolic pathway were over-represented for the differentially expressed genes between these groups. A DNA methylation analysis in promoters of differentially expressed genes indicated a significant difference in DNA methylation at the *IYD* gene. If these responses on carcass traits, gene expression and DNA methylation withstand verification and can indeed be attributed to transgenerational epigenetic inheritance, it would open up pioneering application in pork production and would have implications for human health.

## Introduction

There is a growing body of evidence that environmental effects including nutrition affect the epigenetic code in mammals and that such induced modifications are transmitted to next generations [1], [2], [3]. Transgenerational epigenetic inheritance is defined as metastable epimutations induced by environmental effects that are transmitted to next generations. In a gestating female that was exposed to an environmental trigger only in F3 individuals epigenetic transgenerational inheritance can be established [4]. This is because in a gestating F0 female the F1 embryo or fetus and its germ cells (future F2 generation) are also directly exposed to the environmental effect. Correspondingly, in the male line the F0 male and his germline which potentially produce the F1 generation are exposed to an environmental influence and thus the F2 is the first generation, which was not directly exposed to a specific environment [4], [5], [6]. There are still very few and specific examples by which such “Lamarckian” inheritance induced by ancestral environments could be documented. An often quoted example of transmission of epigenetic modifications in this context is the study in viable yellow (A^vy^/a) inbred mice, where the maternal diet affects DNA methylation at a retrotransposon of the agouti locus that persists over two generations [7], [8], [9]. Another outstanding example of stably transmitted epialleles is the murine *Axin fused* (*Axin^Fu^*) allele where the phenotype of a kinked tail is associated with DNA methylation at a retrotransposon within *Axin^Fu^* and can be transmitted through both the maternal and paternal line [10], [11]. Very recently differential hepatic expression involved in lipid and cholesterol biosynthesis was measured in offspring from male mice that were fed a low protein or a control diet. It was observed that DNA methylation in liver was modestly changed at various loci including a likely enhancer for the lipid regulator *Ppara* [12]. In these examples the transmission of epigenetic modifications and their associated phenotypes were demonstrated, however, the unequivocal segregation of an induced epimutation between generations needs to be proven in order to claim true transgenerational epigenetic inheritance. Additional support for the existence of transgenerational inheritance of nutritional effects comes from an epidemiological study using historical data from Överkalix parish, Norrbotten in northern Sweden [13], [14]. The main findings of these studies indicate a nutrition-linked mechanism that segregates from grandfathers to grandsons and affects their risk of cardiovascular and diabetes mellitus morbidity. Our pilot pig study to investigate the segregation of nutritional effects in a three generation pig pedigree was inspired by the Överklix study and the diet was adapted from that fed to pregnant agouti mice [13], [15]. We hypothesized that environmental perturbations such as dietary modifications affects the epigenetic code in pigs which is transmitted up to two generations later. For this purpose F0 boars were fed a diet enriched with methyl donors or a control diet to investigate heritable epigenetic effects in F2 offspring phenotypes, which would be truly transgenerational in nature. This implies that effects of ancestral F0 boars' diet are transmitted over two generations and thus circumvent epigenetic reprogramming in gametogenesis and early embryogenesis [16]. Here we report the results of nutritional effects on carcass traits in F2 offspring derived from the differentially fed F0 boars and a micro-array based gene expression analysis in liver, muscle and kidney tissues of F2 pigs. We further present a pathway analysis of microarray gene expression data and studied DNA methylation in promoter regions of differentially expressed genes.

## Results

### Three Generation Pig Feeding Experiment

The micronutrients added to the diet E were selected based on the one-carbon metabolism involved in DNA methylation. Due to the lack of information on the potential outcome of the study we decided to feed the boars a combination of all micronutrients in elevated concentrations. The enriched diet was assumed to cause no harm to the F0 boars. After 4 months of age several E boars frequently showed symptoms of neurotoxicity [17]. It was hypothesized that the problems were caused by the high dosage of the potentially neurotoxic vitamin B_6_ [17]. In the following generations, pigs originating from the F0 E boars did not show any of the aforementioned signs.

### Carcass Traits

As expected, carcass traits were affected (*P*<0.05) by gender (Table 1). Barrows had a lower lean meat, loin, and ham percentage but higher adipose tissue, fat of the ham, and fat of the shoulder percentages compared to female pigs (data not shown). These results were expected and not the focus of this study since it is well established that barrows are usually fatter than gilts at a slaughter weight around 105 kg.

**Table 1:** Estimates of effects on carcass traits of F2 offspring from differentially fed F0 boars.

Effect	Shoulder (%)		Backfat (%)		Adipose tissue (%)		Fat thickness at the 10^th^ rib (mm)		Fat thickness at the croup (mm)	
	Est.	Error	Est.	Error	Est.	Error	Est.	Error	Est.	Error
Diet, C	12.25	0.12	8.10	0.24	13.61	0.35	17.74	0.90	21.00	0.89
Diet, E	12.60	0.12	7.27	0.21	12.74	0.31	15.36	0.80	18.70	0.80
*P*	0.066		0.027		0.093		0.076		0.086	
Sex, b	12.33	0.10	8.17	0.24	13.95	0.27	17.29	0.69	19.86	0.67
Sex, f	12.52	0.10	7.22	0.21	12.39	0.26	15.81	0.75	19.83	0.72
*P*	0.082		<0.001		<0.001		0.069		0.965	
Diet×sex c×b	12.15	0.15	8.59	0.27	14.46	0.40	18.71	1.05	20.65	1.02
c×f	12.35	0.16	7.63	0.29	12.39	0.41	16.78	1.10	21.34	1.07
e×b	12.51	0.13	7.74	0.24	13.44	0.34	15.86	0.89	19.08	0.88
e×f	12.70	0.13	6.80	0.25	12.39	0.36	14.85	1.01	18.32	0.98
*P*	0.950		0.957		0.582		0.562		0.323	

c: control diet, e: experimental diet, b: barrow, f: female, Est.: Estimate of the effect.

Compared to F2 C offspring, carcasses of F2 E offspring had lower (*P* = 0.03) backfat percentage, thinner (*P* = 0.09) 10^th^ rib and croup backfat and higher (*P* = 0.07) shoulder percentage (Table 1).

### Gene Expression Profiling

**Figure 1:**
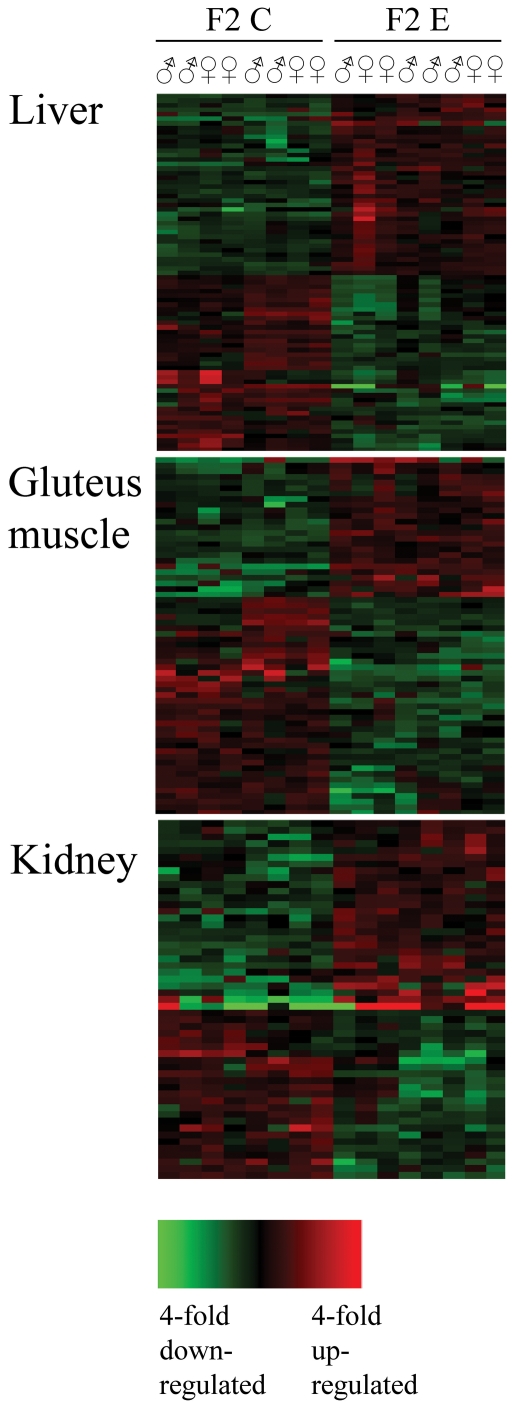
Clustering heat maps for the gene expression analysis. Clustering heat maps for the gene expression analysis in gluteus muscle (GM), liver and kidney of F2 control (C) and F2 experimental (E) pigs are shown. The clustering of significant genes (significance threshold 0.01 and log2 Ratio threshold 1(FC>2)) for GM, liver and kidney with respective 79, 64 and 53 genes are shown. The sex of the pigs and their grouping is given on the top of the heat map of GM for all tissues. The color key of fold change is indicated.

Microarray gene expression profiling was performed in order to compare gene expression levels in the GM, liver and kidney tissue between F2 descendants from the experimental F0 boars and the control F0 boars. From the total of represented 43′803 probes on the porcine microarray chip 25′659 (59%), 28′848 (66%) and 33′638 (77%) were found to be present for the GM, liver and kidney RNA, respectively (detection *P*<0.05). We found twofold differences (*P*<0.01) in mRNA levels between the two groups for 79, 64 and 53 genes for GM, liver and kidney RNA, respectively (t-test statistics, *P*<0.01). In Figure 1 the heat blots for the expression analysis in liver, GM and kidney of these differentially expressed genes are shown. A numeric overview of the transcriptome analysis in the 3 tissue samples of F2 pigs is given in Table 2. The detailed results of the expression analysis in GM, liver and kidney discussed in this publication have been deposited in NCBI's Gene Expression Omnibus [18] and are accessible through GEO Series accession number GSE32412 (http://www.ncbi.nlm.nih.gov/geo/query/acc.cgi?acc=GSE32412). The differences between the expression profiles of the 2 groups were statistically significant and thus worthwhile for further investigations.

**Table 2:** Probe counts by significance and fold-change (fc) in the gluteus muscle, liver and kidney.

*P*-value	#significants	FDR	fc≥1	fc≥1.5	fc≥2	fc≥3	fc≥4	fc≥8	fc≥10
				Gluteus muscle					
*P*<0.1	6121	0.41	6121	784	145	36	12	0	0
*P*<0.01	1370	0.18	1370	378	79	13	3	0	0
*P*<0.001	322	0.08	322	126	39	5	2	0	0
*P*<1e-04	74	0.03	74	39	19	3	2	0	0
*P*<1e-05	13	0.02	13	5	4	1	0	0	0
				Liver					
*P*<0.1	4083	0.67	4083	512	122	29	14	2	2
*P*<0.01	608	0.45	608	222	64	19	8	0	0
*P*<0.001	108	0.25	108	58	22	11	6	0	0
*P*<1e-04	24	0.09	24	20	10	5	2	0	0
*P*<1e-05	10	0.03	10	9	5	2	0	0	0
				Kidney					
*P*<0.1	2522	1.00	2522	414	123	40	18	3	3
*P*<0.01	347	0.94	347	143	53	19	7	2	2
*P*<0.001	57	0.55	57	39	17	7	0	0	0
*P*<1e-04	13	0.25	13	9	5	3	0	0	0
*P*<1e-05	5	0.04	5	4	4	3	0	0	0

# significants: Number of significant probes that differ between the two groups on the significance level indicated.

FDR: False discovery rate.

### Pathway Analysis of Differentially Expressed Genes

We performed a pathway analysis of individual microarray gene expression data of, GM, liver and kidney. Differentially expressed genes that had a *P*-value of less than 0.01 were filtered and used for the pathway analysis. The dataset of GM, liver and kidney contained 1370, 608 and 347 genes, respectively, which were mapped to 313, 109 and 45 human orthologous genes, respectively (Table S1, Table S2, Table S3). These genes were used for the pathway analysis. In liver we found significant pathways (*P*<1.26e-11) that revolved around the transcription factor v-myc myelocytomatosis viral oncogene homolog (c-Myc or MYC) (Figure S1). The involved pathways are given in Table S4 and include transcription initiation and elongation. In GM we found a significant network around hepatocyte nuclear factor 4 alpha (HNF4A) including pathways of phosphorylation and regulation of TOR (*P*<9.85e-62) (Figure S2, Table S1). Metabolic process was significantly associated with differential gene expression in GM (*P*<3.71e-12). The most significant process that matches the liver expression data were the cellular lipid metabolic process (*P*<2.61e-08). The pathway analysis of the filtered kidney dataset indicated also pathways connected to c-Myc involving regulation of cell proliferation and response to wounding (Figure S3, Table S4). In Table S5 the processes that are highly associated with the gene expression data of GM, liver and kidney are listed. It remains elusive of how to relate regulation of cell proliferation and response to wounding from the analysis of kidney gene expression data to the methyl-supplemented diet of F0 boars. In contrast, the lipid metabolism and metabolic pathway obtained from the analysis of liver and GM gene expression data, respectively, fit perfectly to the observed phenotypic differences in fat traits between the C F2 and E F2 offspring.

### DNA Methylation Analysis

**Figure 2:**
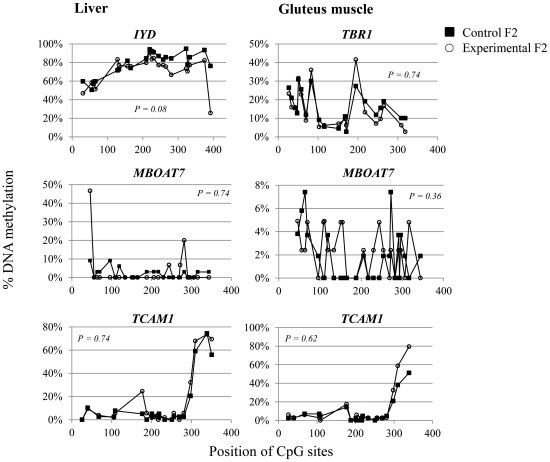
DNA methylation profiles at *IYD*, *TBR1*, *MBOAT7* and *TCAM1* are shown. The DNA methylation content at each CpG site is indicated in the promoter regions of *IYD*, *TBR1*, *MBOAT7* and *TCAM1* in liver and gluteus medius, respectively. The open circles show the results for the control (C) F2 group and the black squares show the results for the F2 experimental (E) F2 group.

Based on our hypothesis that the diet affects the epigenome of future generations in pig we performed a DNA methylation analysis. We selected *TBR1* and *IYD* genes that were differentially expressed in GM and liver of F2 C and E offspring, respectively as well as *MBOAT7* and *TCAM1* that were differentially expressed in both GM and liver of these offspring. Real-time PCR quantification of *IYD* (t-test statistics, *P* = 0.02) and *TCAM1* (t-test statistics, *P*<0.001) expression but not *MBOAT7* (t-test statistics, *P* = 0.79) expression confirmed the microarray gene expression results in liver. However, the trend in *MBOAT7* liver expression was similar between the two methods. In GM *MBOAT7* (t-test statistics, *P* = 0.06) and *TCAM1* (t-test statistics, *P*<0.001) expression was similar to that found in the microarray experiment whereas differential expression of *TBR1* in GM could not be confirmed (Table 3). From each of these 8 F2 C and 8 F2 E offspring DNA methylation in the promoter region of *IYD*, *MBOAT7* and *TCAM1* in liver and *MBOAT7* and *TCAM1* as well as *TBR1* exon1 region in GM was analyzed by clone bisulfite sequencing. This analysis is summarized in Table 4. DNA methylation levels were compared between C F2 and E offspring. The *P*-value resulted from the t-test comparing DNA methylation levels of individual clones between the two F2 groups. A significant difference (*P*<0.05) is indicated between the hypermethylated DNA promoter region in liver of *IYD* clones of 8 C F2 offspring and those of 8 E F2 offspring (Table 4). Higher DNA methylation in the *IYD* promoter is associated with reduced *IYD* expression in C F2 offspring (Table 3). Very low and low DNA methylation levels were found in respective CpG islands of putative promoter regions of *MBOAT7* and *TCAM1* in liver that did not differ between C and E offspring. The DNA methylation analysis yielded similar levels of DNA methylation in the CpG island of *TBR1* exon 1 in muscle between C and E F2 offspring which is in line with *TBR1* gene expression data that could not be confirmed by real-time PCR (Table 3). DNA methylation levels in promoters of *MBOAT7* and *TCAM1* in GM were similar to those in liver and also not different between C and E F2 offspring. The analyzed *MBOAT7* promoter region is hypomethylated in livers of both groups and not associated with gene expression (Table 3). Similar, DNA methylation in the *TCAM1* promoter in both liver and GM was not associated with gene expression. The presented DNA methylation analysis revealed substantial inter-clonal and inter-individual variation in DNA methylation. In Figure 2 the percentage of DNA methylation is shown at each specific CpG site in the promoter region of *IYD*, *MBOAT7* and *TCAM1* and in exon 1 of *TBR1*. The mean values of DNA methylation at each CpG site in the *IYD* promoter region of liver did not differ significantly between C and E F2 offspring (*P* = 0.08). However, the graph in Figure 2 indicates that DNA methylation at *IYD* promoter CpG sites located within the first 200 bp are similar between the two groups whereas significant DNA methylation differences were found at 13 CpG sites between 200 bp and 436 bp of the analyzed *IYD* promoter fragment. No significant CpG methylation differences were observed in the promoter regions of *MBOAT7* and *TCAM1* in liver and muscle. Interestingly, CpG methylation in *TCAM1* differed at the three last CpG sites in muscle but not at the corresponding sites in liver. In *TCAM1* in muscle the result of the last CpG site was excluded from the analysis due to a number of missing values.

**Table 3:** Gene expression resulted from Agilent microarray and real-time PCR between 8 experimental F2 and 8 control F2 pigs.

Gene	Tissue	Normalized Ct value	Real-time PCR, fold difference	Log2 ratio	Microarray analysis, old difference
*IYD*	Liver	0.87	1.82	1.48	2.79
*MBOAT7*	Liver	−0.06	−1.04	−2.05	−4.15
*MBOAT7*	M. gluteus	−0.79	−1.73	−1.52	−2.86
*TCAM1*	Liver	4.06	16.68	1.83	3.56
*TCAM1*	M. gluteus	3.08	8.46	1.38	2.60
*TBR1*	M. gluteus	−0.74	−1.67	2.03	4.07

**Table 4:** DNA methylation was analyzed in six genes of liver and gluteus muscle of 16 F2 pig offspring.

Gene	Product size	Tissue	No. of CpG sites	F2 control (C) (n = no. of clones)	F2 experimental (E) (n = no. of clones)	*P*	Accession No.^1^ Start - end
				Methylated CpG sites	Methylated CpG sites		
*IYD*	436 bp	Liver	21	77% (n = 77)	70% (n = 79)	<0.05	NW_003533902.1 181820–181385
*MBOAT7*	381 bp	Liver	27	3% (n = 33)	2% (n = 15)	0.08	NW_003300106.2 806229–806609
*TCAM1*	373 bp	Liver	21	12% (n = 39)	14% (n = 53)	0.27	NW_003300894.2 74252–74624
*TBR1*	345 bp	Gluteus muscle	20	16% (n = 110)	15% (n = 114)	0.57	NW_003536426.1 385126–384782
*MBOAT7*	381 bp	Gluteus muscle	27	2% (n = 54)	2% (n = 42)	0.31	NW_003300106.2 806229–806609
*TCAM1*	373 bp	Gluteus muscle	21	8% (n = 32)	11% (n = 21)	0.08	NW_003300894.2 74252–74624

1 Sscrofa10 assembly.

We found significant differences in DNA methylation at the *IYD* promoter between C and E F2 offspring which is also associated with gene expression. However, in *TCAM1* in liver and *TCAM1* and *MBOAT7* in GM with confirmed gene expression results we failed to demonstrate any DNA methylation differences. Considering the small sample size and selected genomic regions our DNA methylation results warrant further investigations and more importantly we found indications of differences in the DNA methylation patterns between the two F2 groups of differentially fed F0 boars.

## Discussion

We performed a pilot study to investigate transgenerational response down the male line in a three generation pig pedigree. The hypothesis was that there are differences in phenotypic traits, gene expression and DNA methylation between F2 individuals that could be attributed to the differential feeding of the founder F0 boars. The expected extent and the type of these differences were unknown. Care was taken to have comparable groups of animals concerning the genetic background. The number of individuals was limited. The environment in which all animals were raised was highly controlled and similar for all animals of each generation. We found on each level, gene expression including pathway analysis, DNA methylation and carcass traits, convincing evidence of significant differences between the two groups of F2 offspring. Interestingly, the F2 C offspring tended to be fatter and showed lower percentage of shoulder compared to F2 E offspring. The contributions of individual methylating micronutrients or combinations thereof to the observed effects remain to be established. From our data it could be speculated that micronutrients effectively affected the epigenetic code in F0 boars that was stably transmitted to F2 offspring influencing fat metabolism. However, it is suggested to study less complex mixtures of these methylating micronutrients in a larger group of animals. If these effects on F2 carcass traits hold true it would be a very efficient instrument to manipulate the phenotype of future generations. Furthermore, our results are in line with epidemiological studies in humans demonstrating that environmental exposure affects phenotypic traits of subsequent generations [13], [14]. Also our liver gene expression data including pathway analysis revealed a highly associated cellular lipid metabolic process, which is consistent with the observed nutritional effects on F2 fat traits. In addition metabolic processes were highly associated with gene expression data in GM (Table S5). Overall, gene expression analysis indicated significant differences between the two F2 offspring groups. These differences in gene expression are not trivial to interpret considering the complex architecture of quantitative traits.

A further indication of the existence of transgenerational epigenetic inheritance comes from the DNA methylation analysis. We found DNA methylation differences in the F2 generation that were associated with differential feeding of F0 boars. There were quantitative DNA methylation differences rather than single epimutations at specific CpG sites. Further studies need to establish the causality between DNA methylation relative to gene expression. We found DNA methylation differences in the promoter of the *IYD* gene that may interfere with transcription factor binding. Our DNA methylation analysis was restricted to six promoter regions of differentially expressed genes. Therefore, we suggest that future DNA methylation analysis in such experiments should be performed on a genome wide level, in several individuals and at high coverage due to the high inter- and intra-individual variation in DNA methylation. Nevertheless, single epimutations cannot be excluded. Quantitative DNA methylation patterns were also observed across generations at the murine retrotransposon upstream of the Agouti locus and at a putative Ppara enhancer in F1 mice descendent from low-protein fed F0 male mice [7], [8], [12].

Although we performed our experiment within Swiss Large White breed of balanced genetic background we were not able to separate the genetic from the epigenetic effects. This innate interplay of genetic and epigenetic contributions that model the phenotype was tackled in a plant study investigating descents from a single seed [19]. The authors identified epialleles of DNA methylation that were meiotically stable and heritable across many generations in their *Arabidopsis thaliana* population. Carone et al [12] investigated epigenetic effects of a paternal low-protein diet on the F1 generation in C57/Bl6 inbred mice and observed widespread modest DNA methylation changes between low-protein and control F1 offspring. This finding is in agreement with our DNA methylation study of a small number of selected DNA regions. In the experimental setting of Carone et al. [12] gametogenesis of the founder generation was directly exposed to the diet whereas in our study the focus was on the stable transmission of epigenetic memory up to the F2 generation. The reprogramming of the epigenetic code during primordial germ cell development and/or early embryogenesis argues that all induced DNA methylation changes would be erased and thus not transmitted to the next generation [16]. It is not clear to which extend stable transmission of DNA methylation is attributed to a failure to clear epigenetic marks or other unknown mechanisms. Interestingly, recent results from the Agouti *A^vy^* allele suggest that it is unlikely that DNA methylation is the inherited epigenetic mark [20]. Similarly, DNA methylation differences at the putative mouse *Ppara* enhancer were not reflected in DNA methylation of sperm [12]. Recently it was demonstrated that paramutation-like phenomena induced by RNA molecules were involved in the transmission of phenotypes in mice [21], [22]. The existence of other carriers of heritable information such as RNA and chromatin remains to be established.

In conclusion we found in a three-generation pig feeding experiment phenotypic indications that the F2 generation responded to a methyl-enriched diet exclusively provided to an experimental group of F0 boars. If these responses on carcass traits, gene expression and DNA methylation withstand verification and can indeed be attributed to transgenerational inheritance of epigenetic modifications it would open up new applications in pork production. By this means the efficiency of animal production could be improved and carcasses traits influenced. This study also implies transgenerational response down the male line in humans taking the pig as a model organism. Further comprehensive analyses are necessary to appraise the relevance of transgenerational epigenetic variation in mammals including human.

## Materials and Methods

### Ethics Statement

The study was carried out in strict accordance with the Swiss animal protection legislation. Approval was not necessary since the experiment was considered to cause no harm to the experimental animals.

### Animals and Treatments

**Figure 3:**
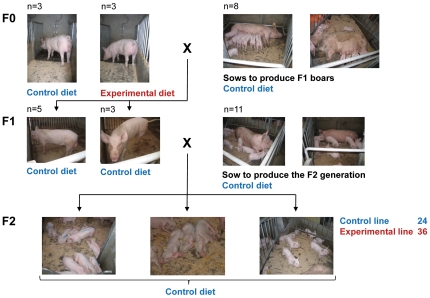
Design of the three generation pig feeding experiment. One group of the F0 boars received a diet enriched with methylating micronutrients (experimental diet E). The F2 offspring were derived from either the F0 boars that received the control diet C or from the F0 boars that received the experimental diet E.

In Figure 3 the design of the three-generation feeding study using Swiss Large White pigs is shown. The study started with 16 F0 boars born from 4 sows, which were mated to 3 different boars. After weaning at d 35 of age, the 16 boars were randomly allotted within litter to 2 feeding groups. Eight experimental boars (**E**) were fed standard starter, grower-finisher and boar diets supplemented with high amounts of methylating micronutrients whereas their control siblings (**C**) were fed standard starter, grower-finisher and boar diets (Table 5). The standard diets were formulated based on the Swiss feeding recommendations for pigs [23]. Out of the 16 boars, 3 E and 3 C boars, 4 of which being littermates, were used to produce the F1 generation. At weaning at 35 d of age, 14 F1 boars (7 originating from F0 E boars [F1 E] and 7 from F0 C boars [F1 C]) were selected and fed the standard C starter, grower-finisher and boar diets as previously described (Table 5). In order to produce the F2 generation, 3 F1 E and 5 F1 C boars were mated to respective 6 and 5 Swiss Large White sows. At weaning at 35 d of age, 36 offspring (F2 E), 16 females and 20 barrows, with a F1 E father and 24 offspring (F2 C), 11 females and 13 barrows, with a F1 C father were selected. All males were castrated within 10 d of birth. The pigs were group-penned and had *ad libitum* access to a standard starter and grower finisher diet as previously described. All pens were equipped with single-space computerized feeders (Mastleistungsprüfung MLP-RAP, Schauer Agrotronic AG, Sursee, Switzerland). During the grower finisher period all animals were periodically weighed and the daily feed intake was monitored.

**Table 5:** Methylating nutrients contents of the diets fed the F0 boars (per kg diet).

	Control diet				Experimental diet			
	Starter	Grower	Finisher	Boar	Starter	Grower	Finisher	Boar
Age, months	1–2.5	2.5–4	4–5	5–10	1–2.5	2.5–4	4–5	5–10
Methionine, g	4.4	3.3	2.5	3.3	11.6	8.5	6.6	8.5
Cysteine, g	3.3	3.5	3.2	2.9	3.2	3.4	3.2	2.9
Choline, mg	300		200		1300		1400	
Betaine, mg	0		0		1600		1600	
Vit. B6, mg	4		3		1600		1600	
Folate, mg	0.5		0.5		200		200	
Vit. B12, mg	0.02		0.02		8		8	

Animals were slaughtered at an average BW of 105 kg. Feed was withdrawn from the pigs 12 h before they were walked to the abattoir of the research station Agroscope Liebefeld Posieux. At the abattoir, animals were electrically stunned, exsanguinated, scalded, mechanically removing the bristles and eviscerated. The hot carcass weight was determined. Thirty minutes after exsanguination, the carcasses entered the air-chilling system (3°C) for 24 h. Within 30 minutes after exsanguination the gluteus muscle (GM), liver and kidney samples were collected from each carcass, immediately frozen in liquid N and stored at −80°C until analysis.

One day after the animals were slaughtered the left side of each carcass was weighed and dissected according to the meat cutting standards applied by the Swiss Performance testing Station (MLP, Sempach, Switzerland), as described previously [24].

### Methyl donor enriched diet

The content of the methylating nutrients of the experimental diets are given in Table 5. The amount of each component was calculated based on indications given by the NRC [25], [26] and Baker [27].

### Microarray expression analysis

The RNA from the GM, liver and kidney tissue were extracted using the Trizol reagent. The integrity of RNA was confirmed by a Bioanalyzer 2100 (Agilent, Waldbronn, Germany). The RNA was labeled following the protocol of the one-color microarray-based gene expression analysis (Quick Amp Labeling, Agilent Technologies, Basel, Switzerland). The quality and the quantity of labeled RNA were inspected with a NanoDrop ND 1000 (NanoDrop Technologies, Delaware, USA). RNA was hybridized to the porcine gene expression microarray from Agilent Technologies according to standard protocol used at the Functional Genomic Center Zürich. We used the Porcine (V2) Gene Expression Microarray, 4×44K (G2519F). This Gene Expression Microarray contains 43′803 probes. The information to construct the array sourced from RefSeq Release 38, Unigene Release 38, TIGR Release 13, Ensemble release 56, and UCSC mRNA. The array is a 4×44K slide format printed using the Agilent's 60-mer SurePrint technology. The sequence information that was used to design the Porcine (V2) Gene Expression Microarray is available from Agilent (https://earray.chem.agilent.com/earray/). Spot intensities that were obtained from the hybridization of the samples to the probes were extracted from the TIFF images using Agilent Feature Extraction Software 9.5. From the generated TXT files the “gMedianSignal” of the spots was used as raw expression value and further analyzed using R/Bioconductor. Expression values were normalized using quantile normalization and differential expression was computed using a t-test on the log2-transformed signals. In each t-test comparing samples from two conditions, only signals of probes were used that were present in at least one of the conditions. A signal of probe was declared present in a condition if it had a linear signal value above 25 and if the flag “gIsWellAboveBG” generated by the Feature Extraction software was true in at least 50% of the replicates of that condition. False Discovery Rates were computed using the Benjamini-Hochberg method. For this microarray expression experiment, GM, liver and kidney RNA from 8 F2 E and 8 F2 C pigs of both genders were used (Figure 1).

### Pathway analysis

Differentially expressed genes in muscle (GM), liver and kidney tissue that had a *P*-value of less than 0.01 were filtered, mapped to the human orthologues and analyzed using the GeneGO MetaCore pathway analysis (db version 6.2, build 24095, http://www.genego.com/metacore.php). The software interconnected all candidate genes according to published literature-based annotations. Only direct connections between the identified genes were considered. In MetaCore analysis, the statistical significance of networks is indicated by a *P*-value from the Fisher's exact test. The false discovery rate (FDR) is used for multiple testing corrections.

### Verification of gene expression by real-time PCR

From the GM and liver samples RNA was extracted using Trizol reagent (Invitrogen, Basel, Switzerland) according to the manufacturers' protocols. RNA was DNase treated according to the supplier's recommendation (Ambion, Rotkreuz, Switzerland). We selected *IYD* (iodotyrosine deiodinase, Accession no.: NM_214416) and *TBR1* (T-box, brain 1, Identifier: A_72_P040316) genes that were differentially expressed in GM and liver, respectively as well as *TCAM1* (testicular cell adhesion molecule 1, Accession no.: CX060127) and *MBOAT7* (membrane bound O-acyltransferase domain containing 7, Accession no.: EW387344) that were differentially expressed in both GM and liver based on the gene expression study using the porcine microarray (Agilent Technologies). To confirm the microarray gene expression results we measured gene expression of *IYD*, *MBOAT7*, *TCAM1* and *TBR1* in GM and liver by RT-PCR using SYBR Green according to the recommendations (ABI, Rotkreuz, Switzerland). We also measured gene expression of three housekeeping genes, *GAPDH* (glyceraldehyde-3-phosphate dehydrogenase), *PPIA* (cyclophilin A) and *ACTB* (actin, beta) by the SYBR Green methodology, which were used for normalization. Normalization was done by subtracting the threshold cycling values (Ct values) of the gene of interest (*IYD*, *MBOAT7*, *TCAM1* and *TBR1*) from the average of the Ct values of the three housekeeping genes. In brief, first-strand cDNA was synthesized according to the manufacturer's protocol (First-Strand cDNA Synthesis Kit, GE Healthcare, Glattbrugg, Switzerland) and the reaction was subsequently purified with QIAquick columns (Qiagen, Hombrechtikon, Switzerland). For this verification GM and liver RNA from the previous 8 F2 E and the 8 F2 C pigs of both genders were used. Each sample was analyzed in triplicate. The sequences of the oligonucleotides are shown in Table S6.

### DNA methylation analysis

We analyzed DNA methylation around CpG islands (EBI Tools CpG Blot, http://www.ebi.ac.uk/Tools/emboss/cpgplot/index.html) in putative promoter regions of *IYD*, *MBOAT7* and *TCAM1* in muscle and *MBOAT7* and *TCAM1* as well as in exon1 of *TBR1* in liver of all 8 F2 E and 8 F2 C pigs of both genders. The sequence information was obtained from NCBI build 3.1, based on Sscrofa10 (http://www.ncbi.nlm.nih.gov/projects/genome/guide/pig/) and from the NAGRP animal genome web site with pig genome build 10.2 sequence information. DNA methylation analysis was performed as recently described [28]. DNA was converted with the EpiTect Bisulfite kit according to the supplier's manual (Qiagen). Bisulfiteconversion-based methylation PCR primers were designed with the program Methprimer (http://www.urogene.org/methprimer/index.html). The primer sequences are shown in Table S7. PCR was performed with the Multiplex PCR Master Mix and products from GM and liver were cloned (TOPO TA Cloning Kit, Invitrogen). White colonies were picked diluted in 10 µl TE following 100 µl LB medium, incubated for 30 minutes at 37°C and amplified with the illustra™ TempliPhi amplification kit (GE Healthcare) and sequenced on an ABI 3730 capillary sequencer (Applied Biosystems). Bisulfite sequencing analysis was performed with the programs BiQ Analyzer (http://biqanalyzer.bioinf.mpi-sb.mpg.de/). DNA methylation was compared between the two groups of F2 offspring with differentially fed F0 boars.

### Statistical analysis

The gene expression data measured by SYBR Green RT-PCR and the DNA methylation quantification were analyzed and tested for significant differences between groups by the Student's t-Test (SAS version 9.2).

The effects of diet on carcass traits were analyzed with PROC MIXED of SAS (version 9.2) using the REML statement. The model was as follows:
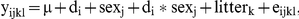
where

y_ijkl_ = carcass trait ijkl,

μ = overall mean,

d_i_ = fixed effect of diet i,

sex_j_ = fixed effect of sex,

d_i_ * sex_j_ = interaction between diet and sex,

litter_k_ = random effect of litter k,

e_ijkl_ = random residual effect of observation ijkl.

## Supporting Information

Figure S1 **Network of pathways that revolved around MYC in liver.** Highly significant pathways that center around the transcription factor v-myc myelocytomatosis viral oncogene homolog (c-Myc or MYC). Up-regulated genes are marked with red circles and down-regulated with blue circles.
(TIFF)

Figure S2 **Significant pathways that centered around HNF4A in gluteus medius (GM).** In GM significant processes revolved around hepatocyte nuclear factor 4 alpha (HNF4A). Up-regulated genes are marked with red circles and down-regulated with blue circles.
(TIFF)

Figure S3 **Network of pathways that revolved around MYC in kidney.** Pathways of processes in revolved in regulation of cell proliferation and response to wounding centered around MYC in kidney. Up-regulated genes are marked with red circles and down-regulated with blue circles.
(TIFF)

Table S1 **Dataset of gluteus muscle mapped to human orthologous genes (XLS).**
(XLSX)

Table S2 **Dataset of liver mapped to human orthologous genes (XLS).**
(XLSX)

Table S3 **Dataset of kidney mapped to human orthologous genes (XLS).**
(XLSX)

Table S4 **Processes that are significantly associated with gene expression data.**
(XLSX)

Table S5 **Top gene ontology processes including** ***P*** **-values that were obtained based on the respective filtered gene expression data.**
(XLSX)

Table S6 **Oligonucleotides used for real-time PCR to quantify gene expression.**
(DOCX)

Table S7 **Oligonucleotides used for DNA methylation analysis using the bisulfite DNA conversion method.**
(DOCX)
