# Supplementary figures and images for: Investigations on Transgenerational Epigenetic Response Down the Male Line in F2 Pigs
Source: PLoS One. 2012 Feb 16;7(2):e30583. doi: 10.1371/journal.pone.0030583 (PMC3281031; doi:10.1371/journal.pone.0030583)

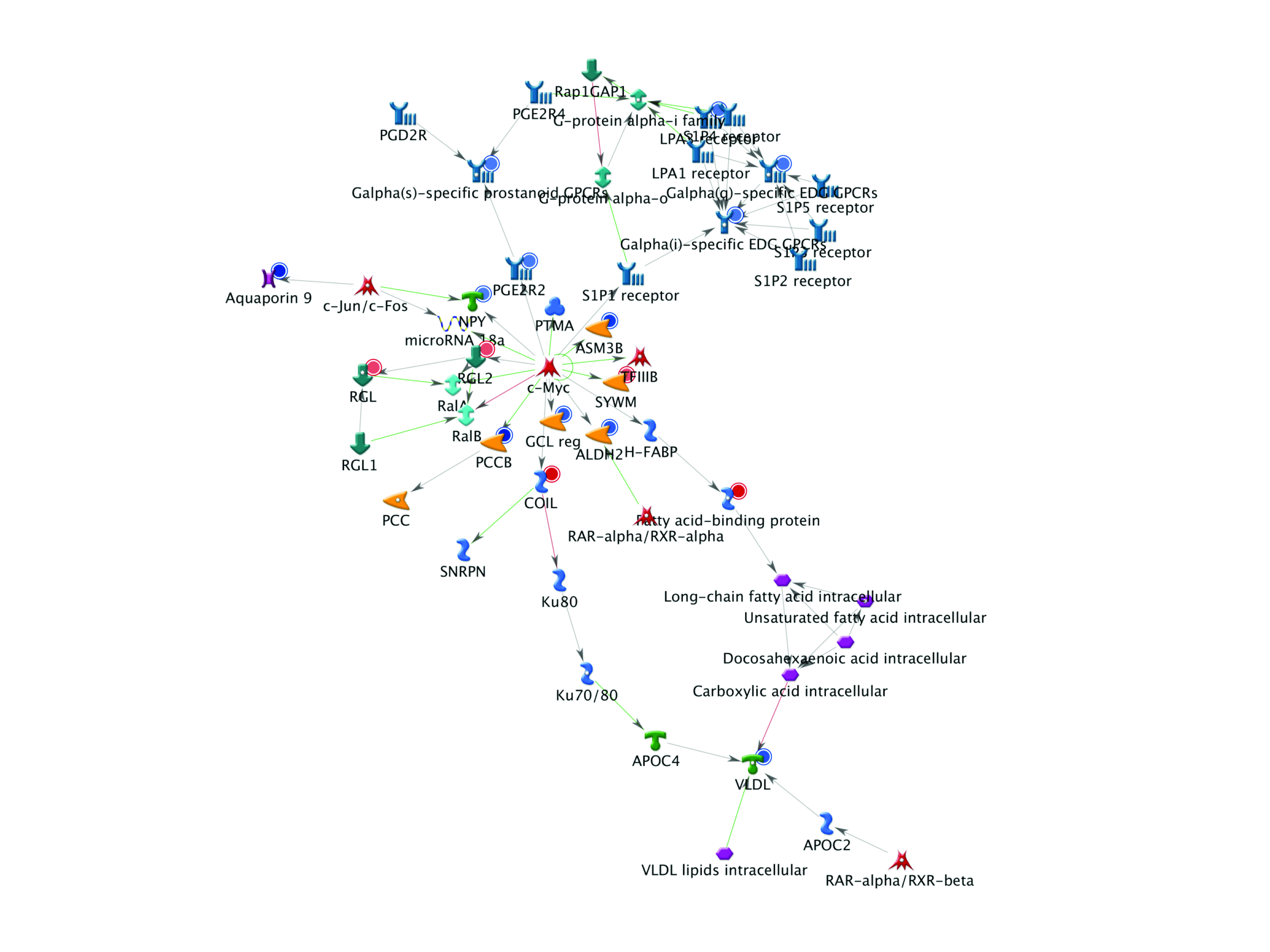

Supplement: Figure S1 — Network of pathways that revolved around MYC in liver. Highly significant pathways that center around the transcription factor v-myc myelocytomatosis viral oncogene homolog (c-Myc or MYC). Up-regulated genes are marked with red circles and down-regulated with blue circles. (TIFF) [file pone.0030583.s001.tiff]

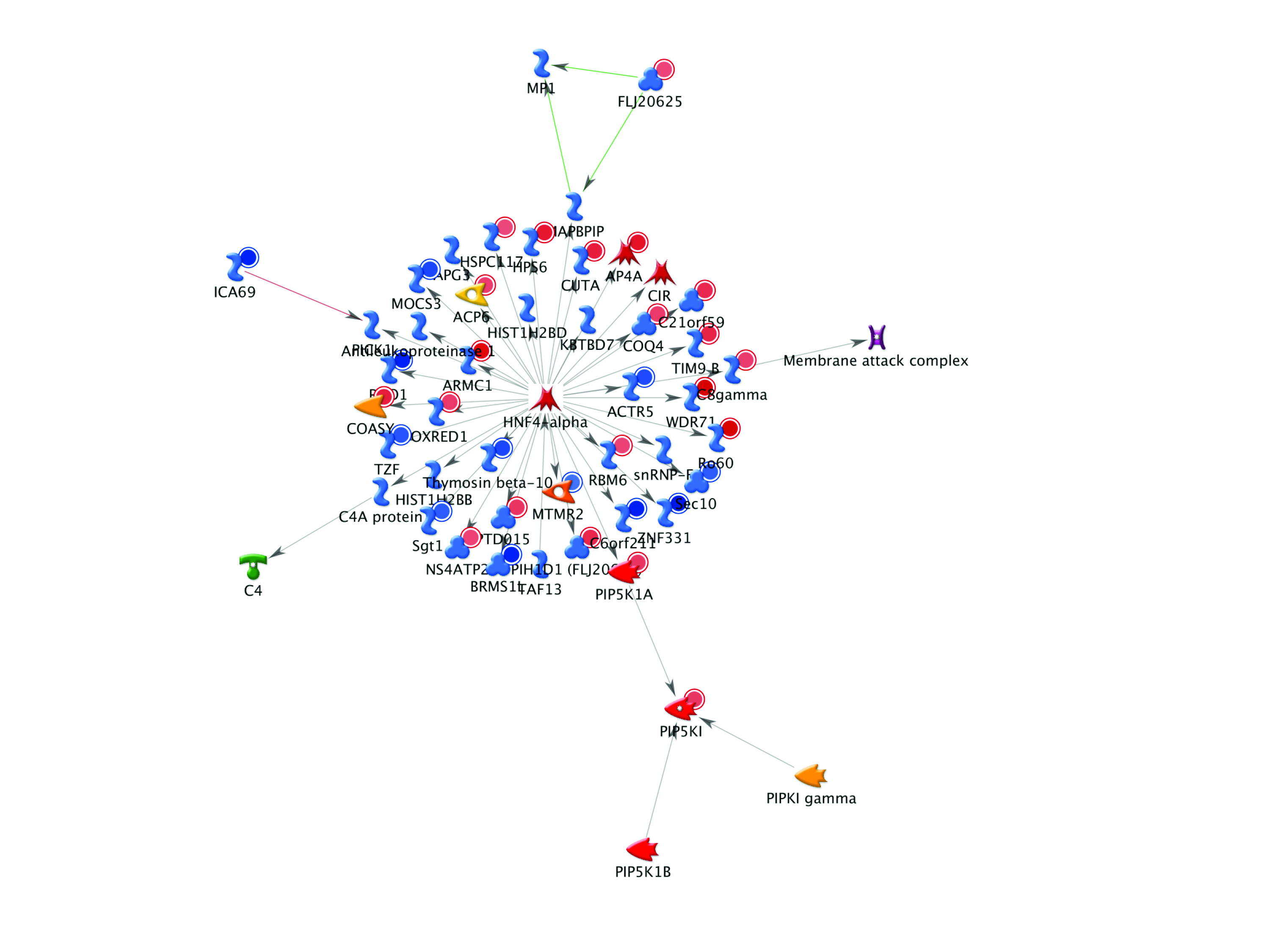

Supplement: Figure S2 — Significant pathways that centered around HNF4A in gluteus medius (GM). In GM significant processes revolved around hepatocyte nuclear factor 4 alpha (HNF4A). Up-regulated genes are marked with red circles and down-regulated with blue circles. (TIFF) [file pone.0030583.s002.tiff]

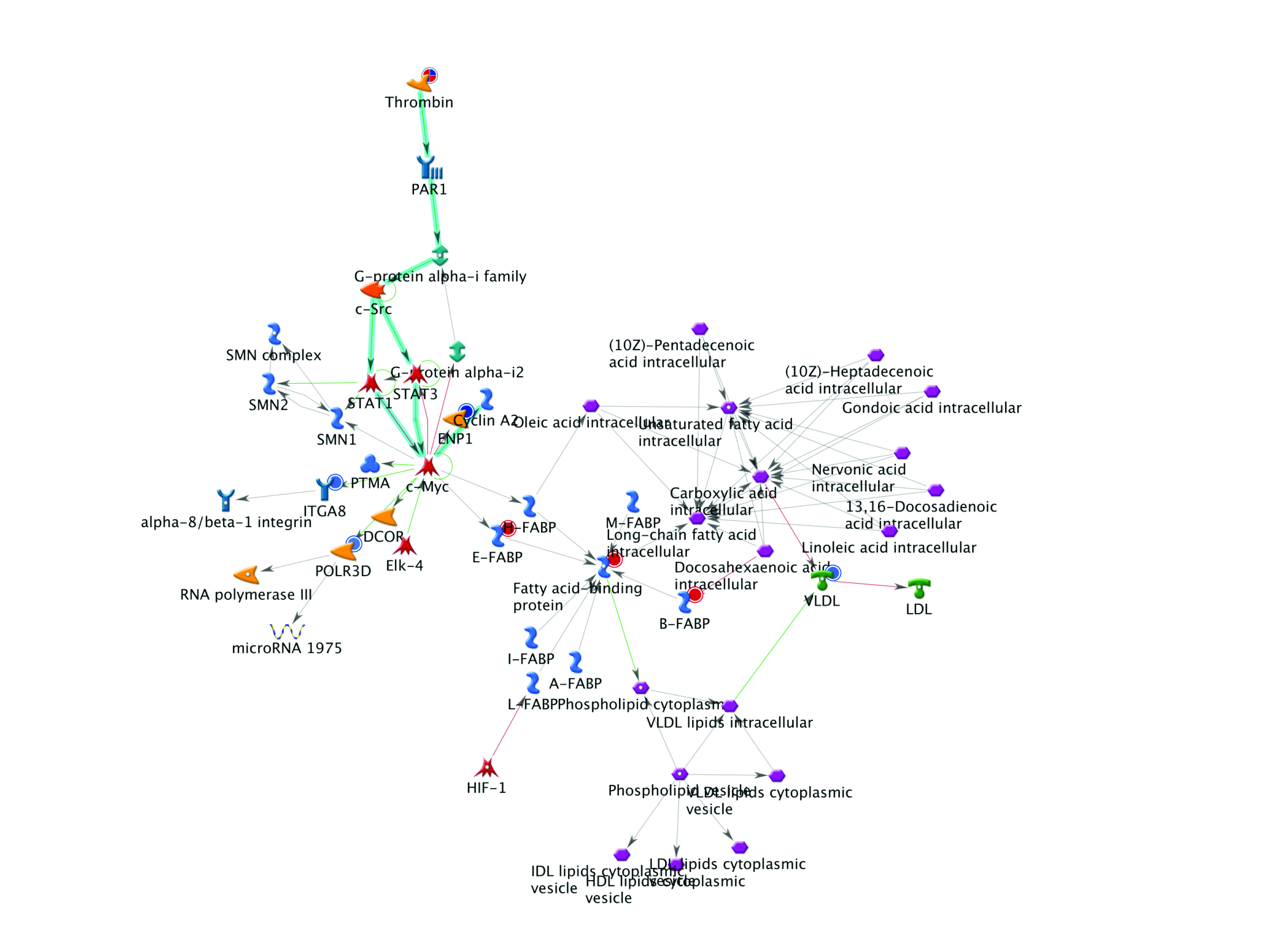

Supplement: Figure S3 — Network of pathways that revolved around MYC in kidney. Pathways of processes in revolved in regulation of cell proliferation and response to wounding centered around MYC in kidney. Up-regulated genes are marked with red circles and down-regulated with blue circles. (TIFF) [file pone.0030583.s003.tiff]
