# Supplementary material for: Investigations on Transgenerational Epigenetic Response Down the Male Line in F2 Pigs
Source: PLoS One. 2012 Feb 16;7(2):e30583. doi: 10.1371/journal.pone.0030583 (PMC3281031; doi:10.1371/journal.pone.0030583)
Supplement: Table S6 — Oligonucleotides used for real-time PCR to quantify gene expression. (DOCX) [file pone.0030583.s009.docx]

**Supplementary Table S6** Processes that are significantly associated with gene expression data.

| Tissue | Processes | P-value |
| --- | --- | --- |
| Liver | Transcription initiation, DNA-dependent (34.0%), transcription initiation from RNA polymerase II promoter (30%), transcription elongation from RNA polymerase II promoter (26.0%), transcription from RNA polymerase II promoter (38%), transcription elongation, DNA-dependent (26.0%) | 1.26e-11 |
| Gluteus muscle | Phosphatidylinositol phosphorylation (8.0%), lipid phosphorylation (8.0%), positive regulation of TOR signaling cascade (6.0%), complement activation, classical pathway (8.0%), regulation of TOR signaling cascade (6.0%) | 9.85e-62 |
| Kidney | Regulation of cell proliferation (54.8%), positive regulation of cell proliferation (41.9%), positive regulation of biological process (67.7%), positive regulation of cellular process (64.5%), response to wounding (41.9%) | 1.42e-13 |
