# Supplementary material for: Investigations on Transgenerational Epigenetic Response Down the Male Line in F2 Pigs
Source: PLoS One. 2012 Feb 16;7(2):e30583. doi: 10.1371/journal.pone.0030583 (PMC3281031; doi:10.1371/journal.pone.0030583)
Supplement: Table S7 — Oligonucleotides used for DNA methylation analysis using the bisulfite DNA conversion method. (DOCX) [file pone.0030583.s010.docx]

**Supplementary Table S7** Top gene ontology processes including *P*-values that were obtained based on the respective filtered gene expression data.

| Tissue | Name of Network | *P*-value |
| --- | --- | --- |
| Liver | cellular lipid metabolic process | 2.61057E-08 |
|  | regulation of catalytic activity | 9.32446E-08 |
|  | regulation of protein catabolic process | 1.63295E-07 |
|  | metabolic process | 2.31392E-07 |
|  | response to stress | 3.01929E-07 |
|  | response to stimulus | 3.19048E-07 |
|  | cellular metabolic process | 3.42914E-07 |
|  | lipid metabolic process | 3.52931E-07 |
|  | regulation of apoptosis | 3.95754E-07 |
|  | cyclic-nucleotide-mediated signaling | 4.05319E-07 |
| Gluteus medius | metabolic process | 3.71036E-12 |
|  | cellular component organization or biogenesis | 1.08245E-11 |
|  | cellular component organization | 1.56885E-11 |
|  | macromolecule metabolic process | 9.43194E-10 |
|  | primary metabolic process | 1.24211E-09 |
|  | organelle organization | 3.31538E-09 |
|  | cellular metabolic process | 3.56117E-09 |
|  | cellular process | 6.7176E-09 |
|  | cellular macromolecule metabolic process | 9.23416E-09 |
|  | protein metabolic process | 2.32814E-08 |
| Kidney | exogenous drug catabolic process | 4.99123E-06 |
|  | drug catabolic process | 8.82036E-06 |
|  | drug metabolic process | 6.87932E-05 |
|  | xenobiotic metabolic process | 0.000158734 |
|  | cellular response to xenobiotic stimulus | 0.000158734 |
|  | response to xenobiotic stimulus | 0.000170323 |
|  | epithelial cell proliferation | 0.00018147 |
|  | monoterpenoid metabolic process | 0.000182093 |
|  | oxidative demethylation | 0.000288765 |
|  | oxidation-reduction process | 0.000526614 |
